# Effects of altitude on thyroid disorders according to Chinese three-rung, ladder-like topography: national cross-sectional study

**DOI:** 10.1186/s12889-023-17569-5

**Published:** 2024-01-02

**Authors:** Boshen Gong, Youmin Wang, Jin-an Zhang, Qiao Zhang, Jiajun Zhao, Jiashu Li, Xichang Wang, Yutong Han, Ziwei Yu, Chenyu Zhang, Bingcong Peng, Yumin Xing, Qiuxian Li, Ping Wang, Yongze Li, Weiping Teng, Zhongyan Shan

**Affiliations:** 1 Department of Endocrinology and Metabolism, Institute of Endocrinology, NHC Key Laboratory of Diagnosis and Treatment of Thyroid Diseases, The First Affiliated Hospital of China Medical University, No. 155, Nanjing Bei Street, Shenyang, Liaoning Province 110001 P. R. China; 2 Department of Endocrinology, The First People’s Hospital of An-Hui Medical University, Hefei, Anhui 230000 P. R. China; 3 Department of Endocrinology, Shanghai University of Medicine & Health Science Affiliated Zhoupu Hospital, Shanghai, 201318 P. R. China; 4 Department of Endocrinology and Metabolism, Guiqian International General Hospital, Guiyang, Guizhou 550004 P. R. China; 5 Department of Endocrinology, Hospital Affiliated With Shandong University, Jinan, Shandong 250012 P. R. China

**Keywords:** High altitude, Geographic variation, Thyroid disorders, Chinese topography, Thyroid antibody

## Abstract

**Background:**

Chinese topography appears a three-rung ladder-like distribution of decreasing elevation from northwest to southeast, which is divided by two sloping edges. Previous studies have reported that prevalence of thyroid diseases differed by altitude, and geographical factors were associated with thyroid disorders. To explore the association between three-rung ladder-like regions and thyroid disorders according to unique Chinese topographic features, we conducted an epidemiological cross-sectional study from 2015–2017 that covered all 31 mainland Chinese provinces.

**Methods:**

A total of 78,470 participants aged ≥ 18 years from a nationally representative cross-sectional study were included. Serum thyroid peroxidase antibody, thyroglobulin antibody, and thyroid-stimulating hormone levels; urine iodine concentration; and thyroid volume were measured. The three-rung ladder-like distribution of decreasing elevation from northwest to southeast in China was categorized into three topographic groups according to elevation: first ladder, > 3000 m above sea level; second ladder, descending from 3000—500 m; and third ladder, descending from 500 m to sea level. The third ladder was further divided into groups A (500–100 m) and B (< 100 m). Associations between geographic factors and thyroid disorders were assessed using linear and binary logistic regression analyses.

**Results:**

Participants in the first ladder group were associated with lower thyroid peroxidase (β = -4.69; *P* = 0.00), thyroglobulin antibody levels (β = -11.08; *P* = 0.01), and the largest thyroid volume (β = 1.74; *P* = 0.00), compared with the other groups. The second ladder group was associated with autoimmune thyroiditis (odds ratio = 1.30, 95% confidence interval [1.18–1.43]) and subclinical hypothyroidism (odds ratio = 0.61, 95%confidence interval [0.57–0.66]) (*P* < 0.05) compared with the first ladder group. Group A (third ladder) (500–100 m) was associated with thyroid nodules and subclinical hypothyroidism (*P* < 0.05). Furthermore, group B (< 100 m) was positively associated with autoimmune thyroiditis, thyroid peroxidase and thyroglobulin antibody positivity, and negatively associated with overt hypothyroidism, subclinical hypothyroidism, and goiter compared with the first ladder group(*P* < 0.05).

**Conclusion:**

We are the first to investigate the association between different ladder regions and thyroid disorders according to unique Chinese topographic features. The prevalence of thyroid disorders varied among the three-rung ladder-like topography groups in China, with the exception of overt hyperthyroidism.

**Supplementary Information:**

The online version contains supplementary material available at 10.1186/s12889-023-17569-5.

## Introduction

**Fig. 1:**
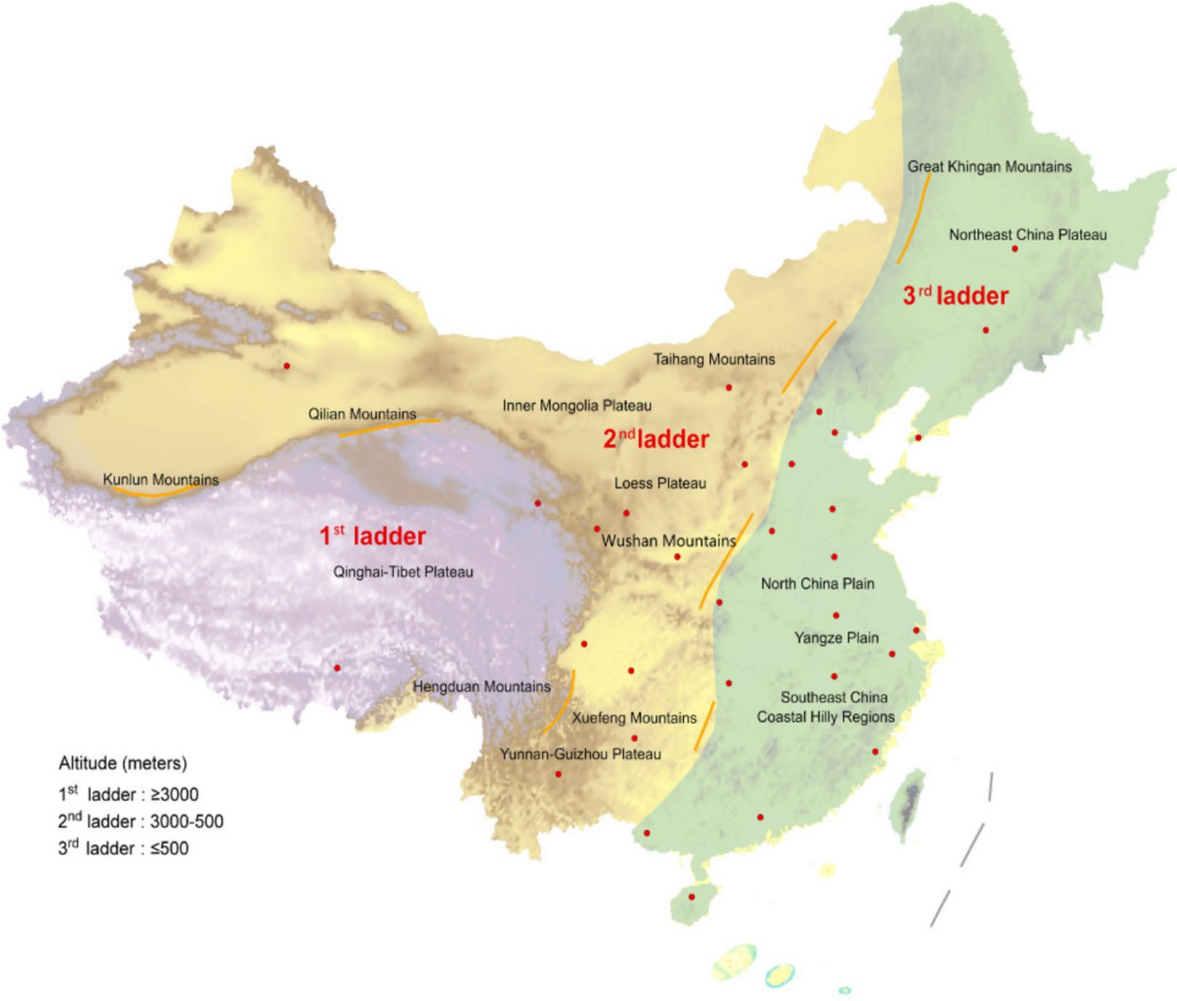
The unique three-rung ladder-like topography of Mainland China. The unique three-rung ladder-like topography of mainland China sloping down from west to east (1st ladder: the first ladder, 2nd ladder: the second ladder, 3rd ladder: the third ladder). Chinese topography was divided into three strata by two sloping edges where two steps meet. The boundary of the first and second ladder regions were Kunlun, Qilian, and Hengduan mountains. The boundary of the third ladder region and second ladder region were Great Khingan, Taihang, Wushan, and Xuefeng mountains

China is located in East Asia on the western shores of the Pacific Ocean. The immense Chinese territory is approximately 9.6 million km^2^. Chinese topography has a recognized three-step ladder-like distribution that slopes downward from northwest to southeast (Fig. 1) [1]. The Kunlun, Qilian, and Hengduan and Great Khingan, Taihang, Wushan, and Xuefeng Mountains are the boundaries for the first and second and second and third ladders, respectively [2]. Over 66% of China consists of upland hills, mountains, and plateaus, and the highest mountains and plateaus are located mainly in the first ladder region, including the highest and largest plateau, the Qinghai-Tibet Plateau, which is known as the “roof of the world” [3]. The second ladder region is mainly comprised of the Inner Mongolia, Loess, and Yunnan-Guizhou Plateaus. The third ladder region is mainly comprised of the Yangtze Plain, Northeast China Plateau, Liaodong Hills, and North China Plain.

Over the past decades, in addition to the traditional factors, many studies focused on environmental factors which have been linked to thyroid diseases, including air pollution and geomorphological features [4–6]. A majority of previous studies found that the prevalence of thyroid diseases varied according to altitude. A cross-sectional survey in Ethiopia reported that altitude was correlated with goiter prevalence, with peak prevalence observed in the highlands [7]. Taylor et al. highlighted geographical differences and the effect of environmental factors on thyroid dysfunction [8]. Furthermore, the microbiota composition of highland residents differs significantly from that of residents at other altitudes, indicating the alteration of the gut-thyroid axis [9, 10]. The hypothesis that geographical factors might be associated with thyroid disorders is reasonable in our opinion. However, there has been a lack of systematic research examining changes in geographic variations related to thyroid diseases prevalence. Also, the geographical areas covered by previous studies were typically small. Therefore, we took advantage of the vast geographical disparity in China to investigate the association between altitude and thyroid disorders in terms of the unique Chinese three-rung, ladder-like topography.

Thyroid hormones play important roles in cell growth and differentiation, and elevated thyroid antibody levels are involved in the progression of many autoimmune thyroid diseases [11, 12]. Several previous studies have reported thyroid status changes at different altitudes [13, 14]. Endocrine system responses to chronic high-altitude exposure were significantly different from those in sea-level areas or low-altitude plain regions. Researchers investigated the thyroid-associated hormones of 1281 participants living in three different altitude regions of China, and found that free triiodothyronine (FT3) increased with altitude, while free thyroxin (FT4) was less influenced [15]. Moreover, the hypothalamic-pituitary-thyroid (HPT) axis was reportedly altered in the high-altitude environments, including hypoxic conditions, hypohydration, and nutrition alterations [16–19].

Recently, a few small, population-based studies explored the association between thyroid status and the first ladder region of Tibet, although these studies did not compare thyroid status with the other two Chinese ladder regions [20]. Here, we used data from the Thyroid disorders, iodine status, and Diabetes, a national epidemiological cross-sectional study (TIDE) conducted from 2015–2017 that covered all 31 mainland Chinese provinces to explore the association between thyroid status and different ladder-like altitude regions, as well as the prevalence of thyroid disorders considering unique Chinese topographic features.

## Materials and methods

### Study design and population

The research protocols were approved by the medical ethics committee of China Medical University (serial number: IRB [2008]115). This study included 80,937 people who were random selected fulfilling the inclusion criteria from urban and rural regions, including all 31 provinces of mainland China. The ration of age and sex urban, or rural population in each region was calculated based on data from the 2010 China census. We used a multiple stratified sampling method to select one representative city per province according to population size and economic level. The inclusion criteria were: 18 years or older, resident of a selected community for at least five years, no iodine-rich drugs such as amiodarone or contrast agents within the previous 3 months, and not pregnant. The exclusion criteria were individuals with missing or invalid data, pre-existing thyroid disorders under medication, a personal history of head or neck surgery or radiation therapy, and current use of drugs that may affect thyroid hormones or thyroid functions. Ultimately, 78,470 participants (38,182 men and 40,288 women) were included in the analysis. This study was approved by the Medical Ethics Committee of the China Medical University. All participants provided written informed consent after we explained all research procedures. Participants did not receive a stipend and all research complied with the declaration of Helsinki. More details of the TIDE study design have been published previously [21].

China’s territory slopes downward from the northwest to southeast similar to a three-step ladder, which is divided by two sloping edges. The first ladder is > 3000 m above sea level (ASL), the second ladder descends from 3000 to 500 m, and the third ladder is < 500 m ASL [22, 23]. Therefore, geographically speaking, we divided all residents into three groups by two sloping edges, where two steps meet (Figure S1). The average altitude of each region was based on the national standards of the People’s Republic of China (GB50009-2012) [24].

### Laboratory tests

Venous blood samples were collected from each participant and transported via a cold chain system to the Central Laboratory in Shenyang for unified testing of thyroid parameters. Serum TSH, thyroid peroxidase antibody (TPOAb), and thyroglobulin antibody (TgAb) levels were measured using an electrochemiluminescence immunoassay on a Cobas 601 analyzer (Roche Diagnostics, Basel, Switzerland) at the Central Laboratory in Shenyang. The sensitivity and specificity of the TSH assay was 0.01 μIU/mL. FT3 and FT4 levels were measured only if TSH levels were outside the reference range (0.27–4.20 mIU/L). The normal ranges for TPOAb, TgAb, and TSH provided by the manufacturers were < 34.00 IU/mL, < 115.00 IU/mL, and 0.27–4.20 mIU/L, respectively. Urine iodine concentration (UIC) was determined using inductively coupled plasma mass spectrometry (Agilent 7700x; Agilent Technologies Inc., Santa Clara, CA, USA). The measurement quality was controlled using certified reference material (GBW09108, GBW9109, and GBW9110) from the Center for Disease Control in China. The target values of the standards GBW09108, GBW9109, and GBW9110 were 70.8 ± 9.0, 143 ± 10, and 224 ± 14 μg/L, respectively; the inter-assay coefficients of variability were 2.3%, 2.5%, and 2.4%, respectively; and the intra-assay coefficients of variability were 2.7%, 1.4%, and 2.3%, respectively.

### Thyroid ultrasonography

All participants underwent thyroid ultrasonography by qualified observers, who had specially trained and passed examination in the project center, using a portable instrument (LOGIQ 100PRO; GE, Milwaukee, WI with a 7.5 MHz linear array transducer). Thyroid volume was calculated as the sum of both lobes volumes estimated according to the formula: V (ml) = 0.479 × width (cm) × depth (cm) × length (cm), without isthmus. Two trained quality control personnel beyond the sonographers were responsible for supervising the accuracy and reliability of the ultrasound results.

### Clinical diagnosis

The diagnostic criteria for thyroid disorders are listed in Table S1.

### Covariates

Demographic information, including age, sex, ethnicity, region of residence, education level, and smoking status, was collected using a standard questionnaire. Ethnicity was categorized as Han or other. Region of residence was identified by questionnaire as living in the selected community for at least 5 years. Education level was categorized as junior school and below or high school and above. Participants who smoked at least one cigarette per day were defined as current smokers. Body mass index (BMI) was calculated according to World Health Organization criteria.

### Quality control

Comprehensive quality control was implemented to ensure the data was valid and well defined. Only investigating staff who passed the performance assessment after training could collect data. Physical examinations were completed by attending physicians, and the instruments were checked and calibrated in time. Members of the steering committee supervised the questionnaire, identified errors, and corrected these in a timely manner. All the data were entered by two persons and verified.

### Statistical analyses

All statistical analyses were performed using SPSS software (version 23.0; IBM Corp., Armonk, NY, USA). We performed a descriptive analysis of BMI, UIC, TPOAb, TgAb, TSH levels, and thyroid volume which were not normally distributed using quartile stratification and compared the medians for differences using the Kruskal-Walls test. The chi-square test was used for categorical data and presented as percentages or counts to compare the thyroid disorder prevalence and related diseases (Table S2). Line chart was used to visually present the results of the prevalence of different thyroid disease among successive three ladder groups. We used linear regression analyses to estimate the TPOAb, TgAb, TSH levels and thyroid volume among the three ladder groups. Linear regression analyses were adjusted for age and sex in model 1 and age, sex, ethnicity, location, education level, smoking status, BMI, and UIC in model 2. Binary logistic regression analyses were adjusted for age and sex in model 1 and age, sex, ethnicity, location, education level, smoking status, BMI, and UIC in model 2, which were used to estimate odds ratios (ORs) and 95% confidence intervals (CIs) and evaluate the risk factors for thyroid disorders and related diseases according to the three ladder groups. The adjusted factors in models were chosen by Direct Acyclic Graph analysis. Statistical significance was defined as *P* < 0.05. Corrected P values were used for pairwise comparisons of multiple groups. Random forests variable importance analysis has been receiving increased attention as a means of variable selection in many studies. Shapley Additive Explanations (SHAP) summary plot is very effective to identify the strength and direction of association between thyroid disorder and its major predictor. The random forest variable importance analysis (Fig S2), and SHAP summary plot analysis (Fig S3) were performed using the RStudio software (version R-4.4.2).

## Results

### Population characteristics according to three-rung, ladder-like topographic groups

Table 1 summarizes the general participant characteristics from the TIDE study by geographic distribution according to the three-rung, ladder-like topographic groups, which revealed that China’s population is mainly concentrated in eastern China, possibly owing to topographic patterns and natural resources [25]. Among 78,470 participants, 60.55% lived in the third ladder region at elevations below 500 m ASL, which was significantly higher than that of the second (32.97%) and first ladder groups (6.47%). People of Han ethnicity was more likely to live in the second and third ladder regions. The median TSH value among participants from the first ladder group was 2.84 mIU/L, which was significantly higher than that in participants from the second ladder (2.51) and the third ladder (2.20) (*P* < 0.05). Moreover, among all three ladder groups, the median thyroid volume in the first ladder group was the largest than the other ladder groups (200.18 μg/L) (*P* < 0.05).

**Table 1:** General characteristics of study population

Altitude (meters)	1st ladder > 3000	2nd ladder 3000–500	3rd ladder < 500	P for difference
Participants n(%)	5080(6.47)	25873(32.97)	47517(60.55)	
Sex				
(M/F)	2265/2815	12711/13162	23206/24311	< 0.001
(M%/F%)	(44.59/55.41)	(49.1/50.9)	(48.8/51.2)	
Age(years)	42.00 (30.00–54.00)	43.00 (30.00–54.00)	43.00 (30.00–55.00)	0.492
Location, n(%)				
Urban	3093(60.89)	13702(53.00)	25065(52.70)	< 0.001
Rural	1987(39.11)	12171(47.00)	22452(47.30)	
Smoke, n(%)				
Yes	1154 (22.72)	7720(29.84)	11745(24.70)	< 0.001
No	3857 (75.93)	18140(70.11)	35710(75.20)	
Education, n(%)				
Junior school and below	3122 (61.46)	12417(47.99)	20353(42.8)	< 0.001
High school and above	1877 (36.95)	13416(51.85)	26872(56.6)	
Ethnicity, n(%)				
Ethnic Han	2645 (52.07)	22006(85.05)	45419(95.6)	< 0.001
Others	2435 (47.93)	3867(14.95)	2098(4.4)	
BMI (kg/m2)	22.89 (20.66–25.78)	23.77* (21.31–26.33)	23.81*(21.36–26.37)	< 0.001
UIC (μg/L)	177.97 (112.17–267.71)	200.18* (135.30–288.40)	170.40* (111.60–253.15)	< 0.001
Thyroid volume	9.76 (7.87–12.07)	7.72* (5.88–10.05)	8.70* (6.58–11.56)	< 0.001
TPOAb (IU/mL)	12.33 (10.11–16.53)	11.38* (7.89–16.71)	9.78* (6.88–14.49)	< 0.001
TgAb (IU/mL)	14.20 (11.51–18.69)	15.79* (12.44–21.40)	14.49 (11.02–19.71)	< 0.001
TSH (mIU/L)	2.84 (1.86–4.47)	2.51* (1.73–3.69)	2.20* (1.53–3.18)	< 0.001

*Abbreviation*: AIT autoimmune thyroiditis, *BMI* body mass index, *SD* standard deviation, *TgAb* thyroglobulin antibodies, *TPOAb* thyroid peroxidase antibodies, *TSH* thyroidstimulatinghormone, *UIC* urinary iodine concentration. 1st ladder: the first ladder, 2nd ladder: the second ladder, 3rd ladder: the second ladder. There were 75 missing values of smoke and 332 missing values of education. The data normality was tested using the Shapiro–Wilk normality test. If data was not normally distributed, the Kruskal–Wallis test followed by a Dunn's multiple comparison test (to compare more than two groups) was used. Group differences for categorical variables were examined using the chi-square test

^*^Compared with the first ladder group, *P* < 0.05

### Associations between ladder regions and clinical thyroid parameters

Table 2 shows the associations between the three ladder regions and TPOAb, TgAb, TSH, and thyroid volume values using linear regression analysis. After adjustments for age, sex, ethnicity, location, education level, smoking status, BMI, and UIC, participants in the second ladder (β = -0.82 [95% CI -0.96 to -0.68] and third ladder (β = -1.24 [95% CI -1.38 to -1.10] were associated with lower serum TSH concentration, compared to the first ladder group. The median TSH value was higher among participants in the first ladder group than that of the other groups (*P* < 0.025). Participants in the second ladder and third ladder group were associated higher TPOAb and TgAb levels compared to the first ladder group after adjustments for confounding factors (*P* < 0.05). Besides, the median thyroid volume among participants in the first ladder group was significantly larger than that in the other ladder groups (*P* < 0.001). As shown in Table 2, thyroid volume was negatively associated with the second ladder group (β = -2.50 [95% CI -2.66 to -2.33]) and the third ladder group (β = -1.37 [95% CI -1.54 to -1.20]) compared to the first ladder group (*P* < 0.05).

**Table 2:** Associations between three-rung ladder-like group and thyroid clinical parameters by linear regression analysis

Groups	TPOAb (IU/mL)				TgAb (IU/mL)				TSH (mIU/L)				Thyroid volume			
	Model 1		Model 2		Model 1		Model 2		Model 1		Model 2		Model 1		Model 2	
	β [CI]	P	β [CI]	p	β [CI]	p	β [CI]	P	β [CI]	P	β [CI]	P	β [CI]	P	β [CI]	p
The 1st ladder	Reference		Reference		Reference		Reference		Reference		Reference		Reference		Reference	
The 2nd ladder	6.47[3.72–3.72–9.21]	< 0.001	5.18[2.31- 8.06]	< 0.001	17.59[8.66–26.52]	< 0.001	15.31[5.91–24.71	< 0.001	-0.74[-0.87- -0.61]	< 0.001	-0.82[-0.96- -0.68]	< 0.001	-2.20[-2.36- -2.04]	< 0.001	-2.50[-2.66- -2.33]	< 0.001
The 3rd ladder	5.75[3.11–8.39]	< 0.001	4.57[1.73–7.42]	< 0.001	11.14[2.55–19.73]	0.01	8.14[-1.18–17.46	0.09	-1.13[-1.26- -1.01]	< 0.001	-1.24[-1.38- -1.10]	< 0.001	-1.06[-1.21- -0.90]	< 0.001	-1.37[-1.54- -1.20]	< 0.001

The linear regression Model 1: Adjusted for age and sex

The linear regression Model 2: Adjusted for age, sex, ethnicity, location, education level, smoking status, BMI, UIC

*Abbreviation*: CI, 95% confidence interval

### Hyperthyroidism

**Fig. 2:**
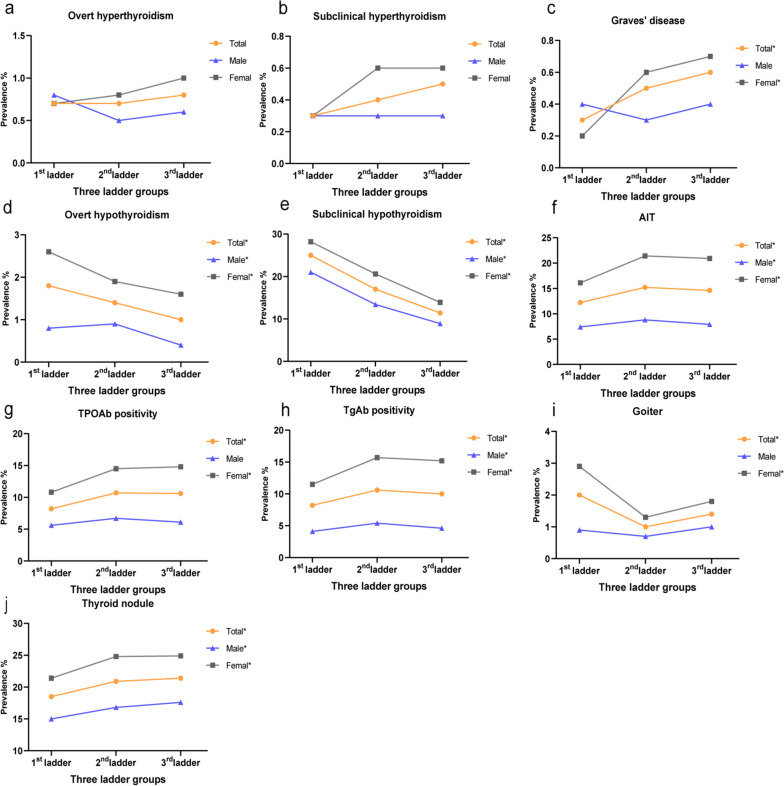
Prevalence of thyroid disorders according to the three-rung ladder-like topography groups. Association of the prevalence of thyroid disorders according to the three-rung ladder-like topography group. **A** Prevalence of overt hyperthyroidism in the overall, male, and female population, stratified by ladder region. **B** Prevalence of subclinical hyperthyroidism in the overall, male, and female population, stratified by ladder region. **C** Prevalence of Graves’ disease in the overall, male, and female population, stratified by ladder region. **D** Prevalence of overt hypothyroidism in the overall, male, and female population, stratified by ladder region. **E** Prevalence of subclinical hypothyroidism in the overall, male, and female population, stratified by ladder region. **F** Prevalence of AIT in the overall, male, and female population, stratified by ladder region. **G** Prevalence of TPOAb positivity in the overall, male, and female population, stratified by ladder region. **H** Prevalence of TgAb positivity in the overall, male, and female population, stratified by ladder region. **I** Prevalence of goiter in the overall, male, and female population, stratified by ladder region. **J** Prevalence of thyroid nodule in the overall, male, and female population, stratified by ladder region. *Chi-square test results compared with the first ladder group, *p* < 0.05/2 = 0.025

No significant association was found between the three successive ladder regions and the prevalence of overt hyperthyroidism and subclinical hyperthyroidism (*P* > 0.05). The prevalence of Graves’ disease (GD) in the first ladder group was lower than that in the other ladder groups (*P* < 0.025) (Fig. 2). Compared with the first ladder group, significant associations were found between the third ladder group (OR = 2.06 [CI = 1.21–3.52]) and GD after adjustments for confounding factors (*P* < 0.05), while no significant difference was found with the second ladder group by binary logistic regression (*P* > 0.05) (Table 3).

**Table 3:** Odds ratio of thyroid disorders among the three-rung ladder-like groups by binary logistic regression analysis

**Altitude**	**The 1st ladder**	**The 2nd ladder**	**The 3rd ladder**
	** > 3000**	**3000–500**	** < 500**
**Overt hyperthyroidism**			
Model1			
OR	Ref	0.95	1.19
CI	Ref	0.66–1.38	0.82–1.71
Model2			
OR	Ref	0.91	1.11
CI	Ref	0.64–1.29	0.79–1.55
**Subclinical hyperthyroidism**			
Model1			
OR	Ref	1.66	1.69
CI	Ref	0.95–2.90	0.98–2.90
Model2			
OR	Ref	2.57^*^	3.00^*^
CI	Ref	1.40–4.74	1.63–5.53
**Graves’ disease**			
Model1			
OR	Ref	1.50	1.85^*^
CI	Ref	0.89–2.54	1.12–3.07
Model2			
OR	Ref	1.65	2.06^*^
CI	Ref	0.96–2.84	1.21–3.52
**Overt hypothyroidism**			
Model1			
OR	Ref	0.77^*^	0.56^*^
CI	Ref	0.61–0.97	0.45–0.70
Model2			
OR	Ref	0.96	0.77^*^
CI	Ref	0.75–1.23	0.60–0.99
**Subclinical hypothyroidism**			
Model1			
OR	Ref	0.62^*^	0.39^*^
CI	Ref	0.58–0.67	0.36–0.42
Model2			
OR	Ref	0.57^*^	0.34^*^
CI	Ref	0.52–0.61	0.31–0.37
**AIT**			
Model1			
OR	Ref	1.35^*^	1.28^*^
CI	Ref	1.23–1.48	1.17–1.40
Model2			
OR	Ref	1.33^*^	1.25^*^
CI	Ref	1.21–1.47	1.14–1.38
**TPOAb positive**			
Model1			
OR	Ref	1.34^*^	1.32^*^
CI	Ref	1.21–1.50	1.19–1.47
Model2			
OR	Ref	1.30^*^	1.28^*^
CI	Ref	1.16–1.46	1.14–1.43
**TGAb positive**			
Model1			
OR	Ref	1.40^*^	1.31^*^
CI	Ref	1.26–1.57	1.18–1.46
Model2			
OR	Ref	1.35^*^	1.24^*^
CI	Ref	1.20–1.51	1.10–1.39
**Goiter**			
Model1			
OR	Ref	0.50^*^	0.69^*^
CI	Ref	0.39–0.62	0.56–0.85
Model2			
OR	Ref	0.56^*^	0.85
CI	Ref	0.44–0.71	0.66–1.08
**Thyroid nodule**			
Model1			
OR	Ref	1.17^*^	1.21^*^
CI	Ref	1.08–1.27	1.12–1.31
Model2			
OR	Ref	1.43^*^	1.58^*^
CI	Ref	1.32–1.56	1.45–1.72

Binary logistic regression Model: Adjusted for age, sex, ethnicity, location, education level, smoking status, BMI, UIC. CI, 95% confidence interval, *Compared with the first ladder group (*P* < 0.05). (A) Binary logistic regression analyses of overt hyperthyroidism. (B) Binary logistic regression analyses of subclinical hyperthyroidism. (C) Binary logistic regression analyses of Graves’ disease. (D) Binary logistic regression analyses of overt hypothyroidism. (E) Binary logistic regression analyses of subclinical hypothyroidism. (F) Binary logistic regression analyses of AIT. (G) Binary logistic regression analyses of TPOAb positivity. (H) Binary logistic regression analyses of TgAb positivity. (I) Binary logistic regression analyses of goiter. (J) Binary logistic regression analyses of thyroid nodule

### Hypothyroidism

Both overt hypothyroidism and subclinical hypothyroidism prevalence decreased with descending altitude from the first to the third ladder regions. Participants in the first ladder group had a significantly higher overt hypothyroidism prevalence (1.83%) than that in the second ladder group (1.40%), and the third ladder group (1.02%) (*P* < 0.001) (Fig. 2). Compared with the first ladder group, the third ladder showed a significant association with overt hypothyroidism (OR = 0.77 [CI = 0.60–0.99]) (*P* < 0.05). However, no significant association was found between the second ladder group and overt hypothyroidism (*P* > 0.05) (Table 3). Participants in the first ladder group had a higher prevalence of subclinical hypothyroidism than that in the other ladder groups (*P* < 0.001). After adjustments for confounding factors, the OR of the second ladder group was 0.57 [CI 0.52–0.61], and that of the third ladder was 0.34 [CI 0.31–0.31], compared with that of the first ladder group.

### Autoimmune thyroiditis

The autoimmune thyroiditis (AIT) prevalence and TPOAb and TgAb positivity increased with descending altitude. The AIT prevalence was 12.20%, 15.21%, and 14.55% for the first ladder group to the third ladder group. The prevalence of TPOAb and TgAb positivity in the second ladder group and the third ladder group were higher than that in the first ladder group (all *P* < 0.001) (Fig. 2). After adjustments for confounding factors, the ORs [CIs] of AIT in the second ladder group and the third ladder group were 1.33 [CI = 1.21–1.47], 1.25 [CI = 1.14–1.38], respectively, compared with that of the first ladder group (*P* < 0.05). Furthermore, compared with the first ladder group, the second ladder group showed a significant association with TPOAb (OR = 1.30 [CI = 1.16–1.46]) and TgAb positivity (OR = 1.35 [CI = 1.20–1.51]) (*P* < 0.05) (Table 3).

### Goiter

The goiter prevalence of the overall population and women was significantly higher in the first ladder group than that in the second and third ladder groups, along with the prevalence in men (Fig. 2). Compared with the first ladder group, the second ladder group (OR = 0.56 [CI = 0.44–0.71]) showed negative associations with goiter, while no significant association was observed between the third group and goiter prevalence (OR = 0.83 [CI = 0.66–1.08]) (*P* > 0.05) after adjustments for confounding factors (Table 3).

### Thyroid nodule

Figure 2 illustrates a thyroid nodule prevalence of 18.54% in the first ladder group, which was significantly lower than that in the second ladder groups (20.86%) and the third ladder group (21.35%) (*P* < 0.001). The prevalence of thyroid nodule increased with descending altitude from the first to the third ladder regions. Both men and women showed a similar trend. Table 3 shows the binary logistic regression model results which revealed ORs for the second ladder group of 1.43 [CI 1.32–1.56] and 1.58 [CI 1.45–1.72] for the third ladder group, respectively, compared with that for the first ladder group, after adjustments for confounding factors (*P* < 0.05).

## Discussion

Thyroid disease is a prevalent health problem with potential health consequences with increasing prevalence worldwide [8]. Thyroid disease can occur alone or in the context of other autoimmune diseases such as celiac disease and type 1 diabetes mellitus [26, 27]. Undiagnosed or uncontrolled thyroid disease can lead to adverse outcomes, including cardiovascular disease, osteoporosis or fractures, pregnancy problems, and all-cause mortality [28–30]. While the relationship between iodine status and thyroid disorders have been investigated, the differences in topographic conditions and altitude, which may affect thyroid status, still need to be clarified [21, 31]. Emerging evidence suggests that the gut-thyroid axis plays a significant role in maintaining metabolic and immunological homeostasis in vivo [32]. Notably, residents living at high altitudes have a specific flora enriched in butyrate-producing bacteria [9]. The variations in thyroid disorders might be correlated to the differences in geographical factors, while the results of previous studies were not conclusive and typically covered small geographical areas. To the best of our knowledge, this is the first cross-sectional study with a large sample size and spatial scale of China investigating the association between three ladder regions with different altitudes and thyroid disorders according to unique Chinese three-rung, ladder-like topography.

Here, we found that China’s population distribution was mainly concentrated in eastern China, and fewer people lived in the first ladder region at extremely high altitudes. As the famous “Hu Line” set by Chinese geographer Hu Huanyong described, the region southwest of the Hu Line accounts for 36% of China’s total land mass although 96 percent of its total population [2]. Moreover, among these three successive ladder regions, people of Han ethnicity mainly lived in the second and third ladder regions. The thyroid disorder prevalence differed significantly among the three ladder regions of China (*P* < 0.05). We found that the prevalence of GD, AIT, TPOAb positivity, TgAb positivity, and thyroid nodule increased while the overt hypothyroidism, subclinical hypothyroidism, and goiter prevalence decreased with elevation descending from the first to third ladder regions. After adjusting for confounding factors, significant associations were found between the three-rung, ladder-like regions and thyroid disorders, whereas no significant association was found between the three successive ladder regions and overt hyperthyroidism and subclinical hyperthyroidism prevalence.

GD is one of the most common causes of hyperthyroidism [33]. We found no significant association between the three ladder regions and overt hyperthyroidism and subclinical hyperthyroidism (*P* > 0.05). The third ladder group showed a positive association with GD compared with the first ladder group. Study have pointed out that the diagnosis and treatment of GD differs by various geographic area [34]. A Swedish study reported that 75% of patients with hyperthyroidism had GD, and geographic differences were observed [35]. Taylor et al. [8] reviewed the global hyperthyroidism incidence and prevalence and highlighted the effects of geographic differences and environmental factors. Considering China’s unique ladder-like terrain, we aimed to describe the hyperthyroidism prevalence based on topographic factors. Several studies explored the thyroid status and thyroid disorder prevalence in Tibet (first ladder region) with a small sample size [15, 20, 36]. Furthermore, they only investigated Tibetan regions without comparisons with the other two ladder regions of China with a limited population. We included all three ladder regions and found that the GD prevalence was significantly lower in the first ladder group than the other ladder groups.

TSH concentration is one of the most sensitive indices of HPT axis function [37]. Studies have shown that the adrenal, thyroid, and gonadal axes are affected by increased altitude, and the HPT axis is altered to adapt to hypoxic conditions [38–40]. Under transient high-altitude exposure, such as mountaineering expeditions, Hackney found significantly lower TSH and FT3 concentrations among 15 mountain climbers who climbed Mount. McKinley in Alaska [41]. Another study conducted at Mount Him lung Himal revealed that the thyroid axis was directly activated by increased altitude and that FT4 increased above baseline at an altitude of 4844 m ASL, although the results for FT3 and TSH were unclear [42]. Animal studies have concluded that high altitude exposure results in a decreased requirement for thyroid hormones and concomitant hormone genesis [43]. A cross-sectional study of long-term high-altitude exposure found that the free thyroid hormone at high altitude is not dependent on the thyroid stimulating hormone released from the anterior pituitary [13]. Notably, the general reference intervals (RI) for thyroid-associated hormones may be not applicable for specific groups, such as the elderly and pregnant women. Similarly, residents living in the first ladder region at a high altitude may have hormone levels inconsistent with those in the plain areas. Thus, diagnosis of thyroid disease among individuals dwelling at high altitudes remains a challenge. Recently, a study investigated thyroid-associated hormones of 1281 participants living in Tibet, the first ladder region of China, and established altitude-specific RI for thyroid-related hormones among residents living in high altitude [15]. They found that FT3 increased with altitude, while FT4 was less influenced by altitude. In our study, TSH levels decreased with descending elevation from high altitude to sea level.

AIT is characterized by thyroid-specific autoantibodies and is one of the most common autoimmune disorders [44]. To date, our study is the first to research the AIT prevalence among the three-rung, ladder-like regions in China. According to unique Chinese geographic features, we found that participants in the first ladder group, with an average altitude above 3000 m, had the lowest prevalence of AIT and thyroid antibody positivity. The diagnosis of AIT mainly relies on circulating antibodies to thyroid antigens (TPOAb and TgAb). The prevalence of AIT and TPOAb and TgAb positivity showed a similar trend, which like a A-shaped curve. Compared with the first ladder group, the other two ladder groups showed a significant association with AIT by binary logistic regression analysis.

A study among schoolchildren in high- and low-altitude areas of Saudi Arabia reported that children living at high altitudes (3150 m ASL) were 2.5 times more likely to develop goiter than their counterparts at low altitudes (500 m ASL) [45]. Another community-based, cross-sectional survey in Ethiopia found that altitude was correlated with goiter prevalence, with peak prevalence observed in the highlands [7]. We also found that the goiter prevalence in the first ladder group was higher than that in other ladder groups, with the lowest prevalence observed in the second ladder group. A meta-analysis of 26 articles assessed the thyroid nodule prevalence in mainland China and found that participants living at elevations below 200 m ASL had a higher prevalence compared with other elevation subgroups [46]. Similarly, we found that the third ladder group had a higher prevalence of thyroid nodules than that of other two ladder groups.

Our study has several strengths. First, we selected participants from all 31 Chinese provinces, including the first through the third ladder regions, which comprehensively revealed the association between thyroid disorders and different altitudes based on Chinese topographic features. These findings may improve our understanding of thyroid disease epidemiology and help guide public health interventions. Second, most previous studies only detected thyroid hormones, including TSH, FT3, and FT4; however, we also detected thyroid volume and thyroid antibodies, including TPOAb and TgAb. Third, previous studies mainly included mountaineers at high altitudes and observed a transient change in thyroid hormone levels [16]. We selected residents from each ladder region who had lived there for at least five years to determine the geographic variations in thyroid status changes. The endocrine changes related to altitude adaptation in humans have long attracted physiologists. However, the underlying mechanism of the altitude effects on thyroid function and diseases is still unclear. Overall, we investigated the association between altitude and thyroid disorders in terms of unique Chinese topographic features, and provided insights and evidence for future study on the correlation between geomorphological factors and thyroid disorders.

This study has several limitations. First, since our study excluded pregnant women and only included adults, the findings are not generalizable for the full distribution of thyroid disorders and thyroid status among the entire Chinese population. Second, environmental factors, such as temperature, humidity, and sunshine duration, as well as iodine intake, genetic and other factors, may influence disease outcomes. Third, selenium is an essential trace element and interacts with other trace elements that together contribute to adequate thyroid hormone status. In our study, we did not evaluate the selenium levels of the population. Furthermore, personal dietary habits such as seafood consumption could be a confounding factor, which may influence thyroid hormone status. Finally, since this was a cross-sectional study, mechanisms involved in the observed phenomena cannot be explained; thus, further studies with larger sample sizes are needed to reveal the mechanism of geographic variation in thyroid status based on the three-rung, ladder-like topography of China.

## Conclusions

In conclusion, we investigated the association between altitude and thyroid disorders according to unique Chinese topographic features providing evidence of the correlation between geographic factors and thyroid disorders and highlight geographic differences and environmental factors in thyroid health research. Owing to the limitations of this cross-sectional study, we did not include individual changes over time; thus, further cohort studies are needed to evaluate temporal changes.

### Supplementary Information

**Additional file 1:** **Supplemental Figure 1.** Flowchart depicting survey design. **Supplemental Figure 2.** Random forest variable importance analysis. **Supplemental Figure 3.** Shapley Additive Explanations Summary Plot analysis. **Supplemental Table 1.** Diagnostic Criteria for Thyroid Disorders. **Supplemental Table 2.** Prevalence of Thyroid Disorders According to the Three-rung Ladder-Like Topography Group.

## Data Availability

The data presented in this study are available on reasonable request from the corresponding author. The data are not publicly available due to reasons of sensitivity of human data.

## Abbreviations

FT3: Free triiodothyronine
FT4: Free thyroxin
HPT: Hypothalamic-pituitary-thyroid
TIDE: Thyroid disorders, iodine status, and Diabetes, a national epidemiological cross-sectional study
ASL: Above sea level
TSH: Thyroid stimulating hormone
TPOAb: Thyroid peroxidase antibody
TgAb: Thyroglobulin antibody
UIC: Urine iodine concentration
BMI: Body mass index
ORs: Odds ratios
CIs: Confidence intervals
SHAP: Shapley Additive Explanations
GD: Graves’ disease
AIT: Autoimmune thyroiditis
