# Supplementary material for: Effects of altitude on thyroid disorders according to Chinese three-rung, ladder-like topography: national cross-sectional study
Source: BMC Public Health. 2024 Jan 2;24:26. doi: 10.1186/s12889-023-17569-5 (PMC10762831; doi:10.1186/s12889-023-17569-5)
Supplement: Supplementary file 1 — Additional file 1: Supplemental Figure 1. Flowchart depicting survey design. Supplemental Figure 2. Random forest variable importance analysis. Supplemental Figure 3. Shapley Additive Explanations Summary Plot analysis. Supplemental Table 1. Diagnostic Criteria for Thyroid Disorders. Supplemental Table 2. Prevalence of Thyroid Disorders According to the Three-rung Ladder-Like Topography Group. [file 12889_2023_17569_MOESM1_ESM.docx]

**
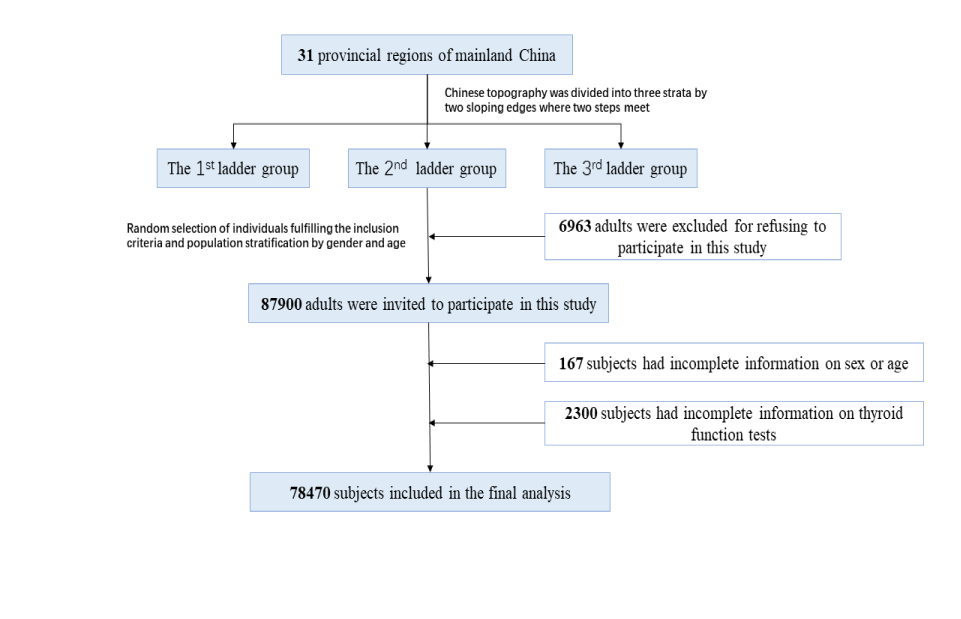
Additional file 1: Supplemental Figure 1. Flowchart depicting survey design**

The entire study included all 31 provinces in mainland China, which divided into three strata, according to Chinese unique three-rung ladder-Like topography. Eligible individuals who met the inclusion criteria were randomly selected and stratified by age and sex. 2,467 participants were excluded because of missing information. Ultimately, 78,470 participants (38,182 men and 40,288 women) were included in the analysis.

**Supplemental Figure 2. Random forest variable importance analysis**


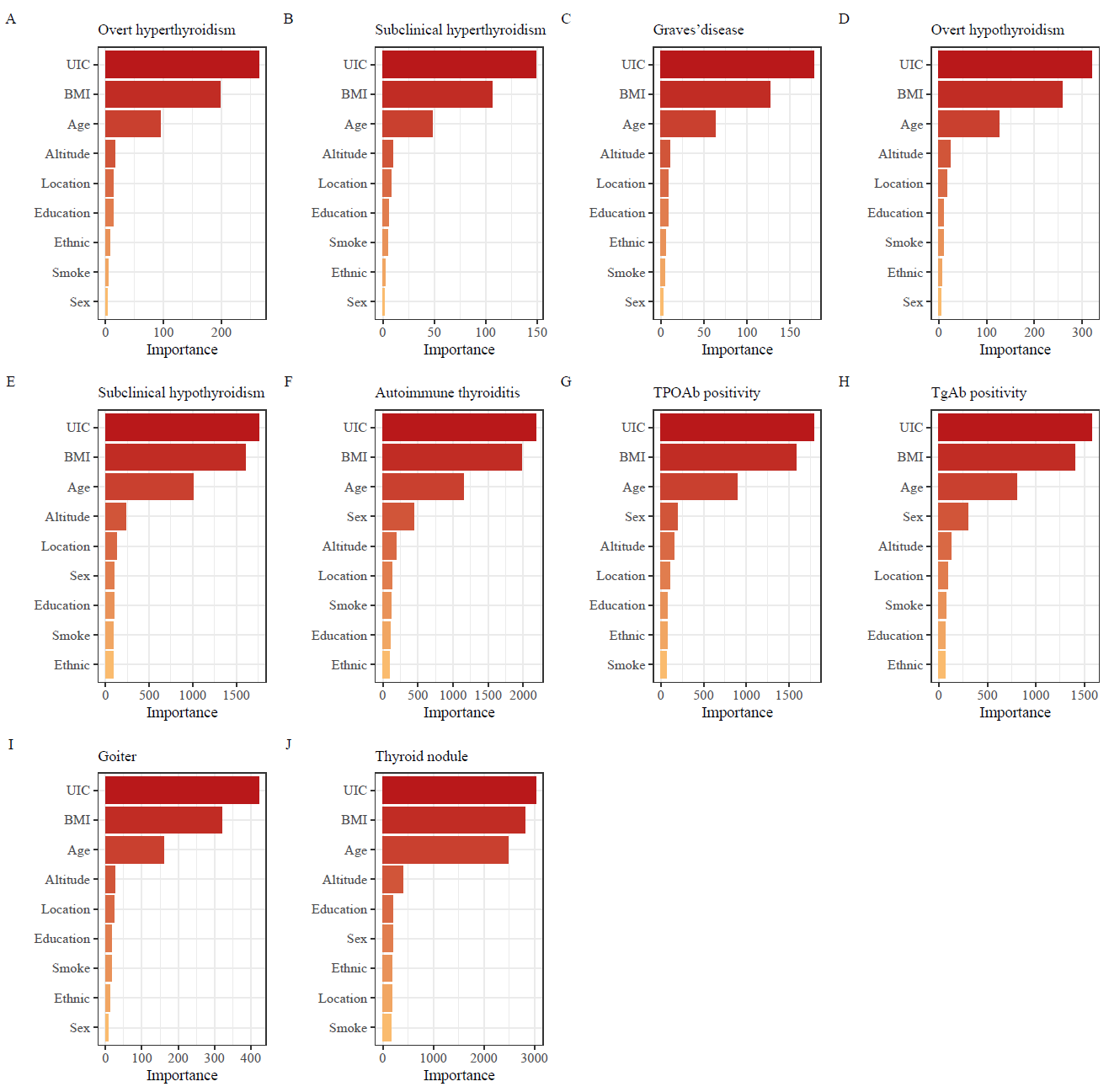


The variable importance plot is based on the Gini index using a random forest approach. (A) Random forest variable importance analysis of overt hyperthyroidism. (B) Random forest variable importance analysis of subclinical hyperthyroidism. (C) Random forest variable importance analysis of Graves’ disease. (D) Random forest variable importance analysis of overt hypothyroidism. (E) Random forest variable importance analysis of subclinical hypothyroidism. (F) Random forest variable importance analysis of autoimmune thyroiditis. (G) Random forest variable importance analysis of TPOAb positivity. (H) Random forest variable importance analysis of TgAb positivity. (I) Random forest variable importance analysis of goiter. (J) Random forest variable importance analysis of thyroid nodule.

**Supplemental Figure 3. Shapley Additive Explanations Summary Plot analysis**


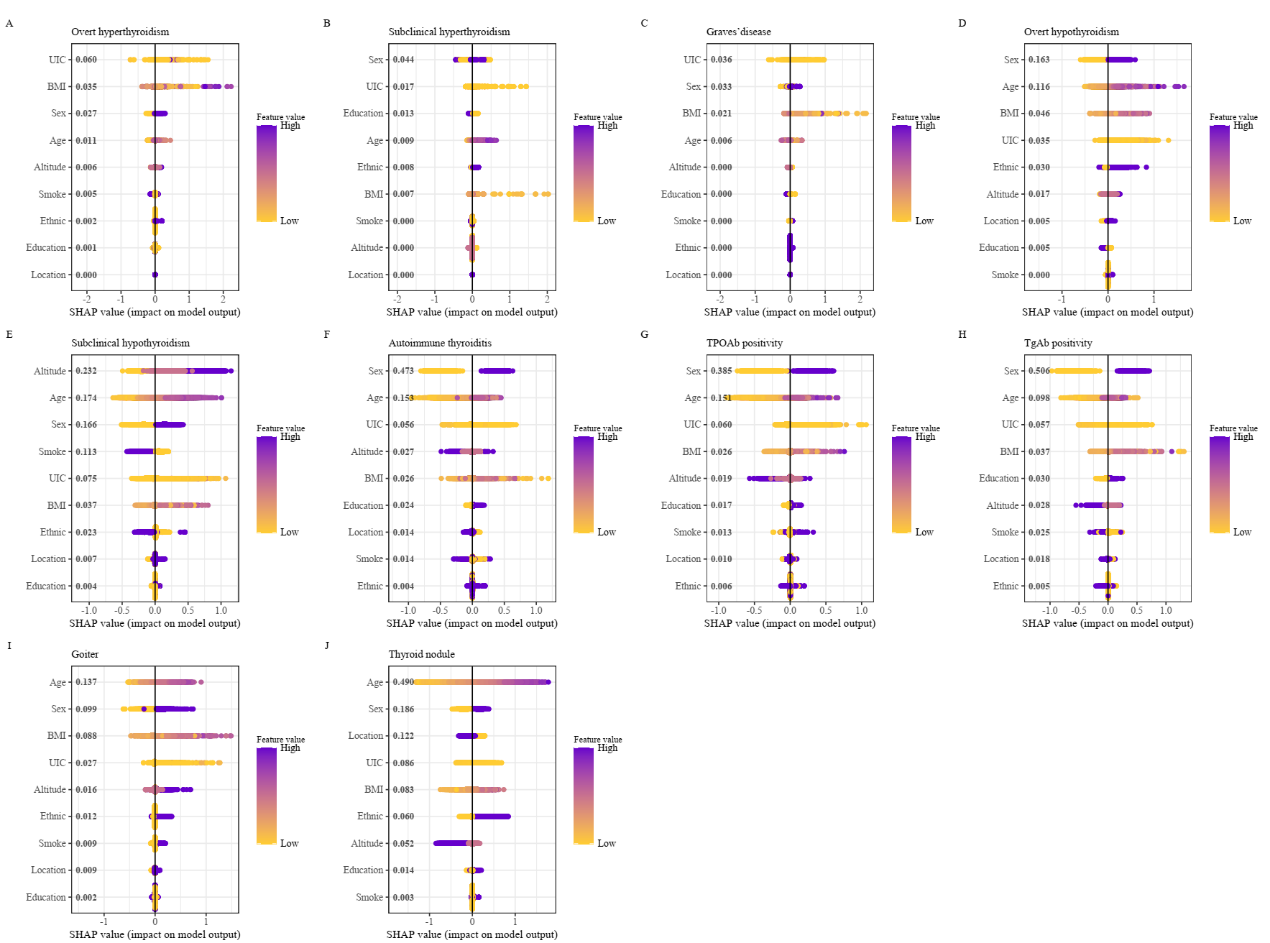


The SHAP summary plot was used to identify the strength and direction of association between thyroid disorders and its major predictors. (A)The SHAP summary plot analysis of overt hyperthyroidism. (B) The SHAP summary plot analysis of subclinical hyperthyroidism. (C) The SHAP summary plot analysis of Graves’ disease. (D) The SHAP summary plot analysis of overt hypothyroidism. (E) The SHAP summary plot analysis of subclinical hypothyroidism. (F) The SHAP summary plot analysis of autoimmune thyroiditis. (G) The SHAP summary plot analysis of TPOAb positivity. (H) The SHAP summary plot analysis of TgAb positivity. (I) The SHAP summary plot analysis of goiter positivity. (J) The SHAP summary plot analysis of thyroid nodule. A dot is created for each feature attribution value for the model of each patient, and thus one patient is allocated one dot on the line for each feature. Dots are colored according to the values of features for the respective patient and accumulate vertically to depict density. Purple represents higher feature values, and yellow represents lower feature values. Abbreviations: BMI, body mass index; TgAb, thyroglobulin antibodies; TPOAb, thyroid peroxidase antibodies.

**Supplemental Table 1. Diagnostic Criteria for Thyroid Disorders**

| **Thyroid disorders** | **Diagnosis criteria** |
| --- | --- |
| Overt hyperthyroidism | TSH <0.27 mIU/L; FT4 >22 pmol/L, or FT3 >6.8pmol/L |
| Subclinical hyperthyroidism | TSH <0.27 mIU/L; FT4 and FT3 within the normal range (FT4 12.0-22.0 pmol/L; FT3 3.1-6.8pmol/L) |
| Graves’ disease | Overt hyperthyroidism or subclinical hyperthyroidism; TRAb >1.75 IU/L or a disffuse goiter on B-mode ultrasonography |
| AIT | TPOAb >34 IU/mL or TgAb >115 IU/mL |
| Overt hypothyroidism | TSH >4.2 mIU/L; FT4 <12 pmol/L |
| Subclinical hypothyroidism | TSH >4.2 mIU/L; FT4 within 12-22 pmol/L |
| TPOAb positive | TPOAb >34 IU/mL |
| TgAb positive | TgAb >115 IU/mL |
| Goiter | Female >22.5 mL; Male >25.4mL |
| Thyroid nodule | One or more nodule (>5 mm) without goiter |

Abbreviations: AIT, autoimmune thyroiditis; TgAb, thyroglobulin antibodies; TPOAb, thyroid peroxidase antibodies；TRAb, thyrotrophin receptor antibody; FT3: Free triiodothyronine; FT4: free thyroxin;

**Supplemental Table 2. Prevalence of Thyroid Disorders According to the Three-rung Ladder-Like Topography Group.**

|  | **1^st^ ladder** | **2^nd^ ladder** | **3^rd^ ladder** | ***P* for difference** | |
| --- | --- | --- | --- | --- | --- |
| **Altitude(meters)** | **>3000** | **1000-2000** | **<500** |  |  |
| Overt hyperthyroidism, n(%)  Male  Female | 38 (0.75)  19(0.84)  19(0.67) | 171 (0.66)  62(0.49)  109(0.83) | 383(0.81)  132(0.57)  251(1.03) | 0.095  0.111  0.047 |  |
| Subclinical hyperthyroidism, n(%)  Male  Femal | 14 (0.28)  6(0.26)  8(0.28) | 116 (0.42)  37(0.29)  79(0.60) | 216(0.45)  70(0.32)  146(0.60) | 0.183  0.947  0.103 |  |
| Graves’ disease, n(%)  Male  Female | 16(0.31)  9(0.40)  7(0.25) | 119(0.46)  42(0.33)  77(0.59) | 269^*^(0.57)  90(0.39)  179^*^(0.74) | 0.019  0.675  0.005 |  |
| Overt hypothyroidism, n(%)  Male  Female | 93 (1.83)  19(0.84)  74(2.63) | 363^*^(1.40)  109(0.86)  254^*^(1.93) | 485^*^(1.02)  98^*^(0.42)  387^*^(1.59) | <0.001  <0.001  <0.001 |  |
| Subclinical hypothyroidism, n(%)  Male  Female | 1269 (24.98)  475(20.97)  794(28.21) | 4406^*^(17.03)  1699^*^(13.37)  2707^*^(20.57) | 5434^*^(11.44)  2064^*^(8.89)  3370^*^(13.86) | <0.001  <0.001  <0.001 |  |
| AIT, n(%)  Male  Female | 620 (12.20)  167(7.37)  453(16.09) | 3936^*^(15.21)  1116(8.78)  2820^*^(21.43) | 6915^*^(14.55)  1824(7.86)  5091^*^(20.94) | <0.001  0.004  <0.001 |  |
| TPOAb positive, n(%)  Male  Female | 429 (8.44)  126(5.56)  303(10.76) | 2764^*^(10.68)  853^*^(6.71)  1911(14.52) | 5016^*^(10.56)  1420^*^(6.12)  3596(14.79) | <0.001  0.030  <0.001 |  |
| TgAb positive, n(%)  Male  Female | 416 (8.19)  92(4.06)  324(11.51) | 2747^*^(10.62)  686^*^(5.40)  2061^*^(15.66) | 4760^*^(10.02)  1064(4.59)  3696^*^(15.20) | <0.001  0.001  <0.001 |  |
| Goiter, n(%)  Male  Female | 104 (2.05)  21(0.93)  83(2.95) | 266^*^(1.03)  90(0.71)  176^*^(1.34) | 671^*^(1.41)  229(0.99)  442^*^(1.82) | <0.001  0.026  <0.001 |  |
| Thyroid nodule, n(%)  Male  Female | 942(18.54)  339(14.97)  603(21.42) | 5398^*^(20.86)  2135(16.80)  3263^*^(24.79) | 10147^*^(21.35)  4092^*^(17.63)  6055^*^(24.91) | <0.001  0.002  <0.001 |  |

Abbreviations: AIT, autoimmune thyroiditis; TgAb, thyroglobulin antibodies; TPOAb, thyroid peroxidase antibodies; *Chi-square test results compared with the first ladder group, *p*<0.05/2=0.025.
